# Haemoglobin decreases in NSAID users over time: an analysis of two large outcome trials

**DOI:** 10.1111/j.1365-2036.2011.04790.x

**Published:** 2011-08-02

**Authors:** J L Goldstein, F K L Chan, A Lanas, C M Wilcox, D Peura, G H Sands, M F Berger, H Nguyen, J M Scheiman

**Affiliations:** * Department of Medicine, University of Illinois at ChicagoChicago, IL, USA; † Department of Medicine and Therapeutics, Prince of Wales HospitalShatin, Hong Kong; ‡ University of Zaragoza, Aragon Health Research Institute (IIS Aragón) CIBERehdSpain; § Division of Gastroenterology/Hepatology, University of Alabama at BirminghamBirmingham, AL, USA; ¶ Division of Gastroenterology and Hepatology, University of Virginia Health Sciences CenterCharlottesville, VA, USA; ** Pfizer IncNew York, NY, USA; †† Division of Gastroenterology, Department of Internal Medicine, University of Michigan Medical SchoolAnn Arbor, MI, USA

## Abstract

**Background:**

Nonsteroidal anti-inflammatory drugs (NSAIDs) have been associated with clinically significant decreases in haemoglobin dependent and independent of acute bleeding events.

**Aim:**

To evaluate the incidence and time to a clinically meaningful decrease in haemoglobin in two double-blind, prospective randomised clinical trials comparing NSAIDs in patients with osteoarthritis (OA) or rheumatoid arthritis (RA).

**Methods:**

In CLASS, patients with OA/RA who were aged ≥18 years and required continuous NSAID treatment were included; patients who were *Helicobacter pylori* positive and/or using aspirin were not excluded. In contrast, in the CONDOR trial, comparing celecoxib alone to diclofenac sustained release (plus omeprazole), patients were aged ≥60 years or ≥18 years with a history of gastroduodenal ulcer and were *H. pylori* negative; aspirin or other anti-platelet users were excluded. To make a parallel *post hoc* analysis we limited our study to 6 months and the populations to only the non-aspirin users in CLASS and those patients receiving either celecoxib or diclofenac. A decrease in haemoglobin of ≥2 g/dL defined the primary end point.

**Results:**

At 6 months, in the CLASS and CONDOR trials, 1.9% and 2.0% of patients treated with celecoxib and 3.3% and 5.7% of patients treated with diclofenac developed a ≥2 g/dL decrease in haemoglobin, respectively, [CLASS: odds ratio (OR) 1.80 (95% confidence interval (CI), 1.22–2.65) and CONDOR: OR 2.93 (95% CI, 2.06–4.15), respectively].

**Conclusion:**

In these two large, independent trials, clinically-meaningful decreases in haemoglobin ≥2 g/dL occurred in a relatively similar fashion over time despite differences in trial designs.

## Introduction

Nonsteroidal anti-inflammatory drugs (NSAIDs) are among the most widely prescribed class of drugs worldwide^1^ because of their anti-inflammatory, anti-pyretic and analgesic properties. However, despite their well-accepted efficacy, chronic use of NSAIDs is associated with an increased risk of gastrointestinal (GI) mucosal injury and overt bleeding events.^2^, ^3^

Previous prospective, randomised outcome studies have focused primarily on GI events in the upper GI tract^4^–^9^ but recent evidence has shown that injury to the small bowel and colon during chronic NSAID use can lead to mucosal damage, ulceration, overt bleeding, obstruction/perforation, protein-loss and occult blood loss (with an associated decrease in haemoglobin).^2^, ^10^–^15^ Extending the understanding of small bowel and colonic injury associated with NSAID use, the Celecoxib vs Omeprazole and Diclofenac for At-risk Osteoarthritis (OA) and Rheumatoid Arthritis (RA) Patients (CONDOR) trial was a prospective randomised outcome trial of 6 months duration that utilised a novel comprehensive composite primary end point of both upper and lower GI events, including clinically significant decreases in haemoglobin (≥2 g/dL).^16^, ^17^ In CONDOR the authors reported that the risk of the adjudicated composite primary end point for clinically significant outcomes throughout the GI tract was lower in patients treated with a cyclo-oxygenase (COX)-2-selective NSAID than in those receiving a nonselective NSAID plus a proton pump inhibitor (PPI). Notably, the difference between the two treatment arms for the primary end point of this trial appeared to be driven by one of the components of the composite end point, the number of patients reaching the predefined cut-off point for a decrease in haemoglobin (≥2 g/dL) and/or haematocrit (≥10%) over the time of exposure to drug.

Like the CONDOR trial, the Celecoxib Long-Term Arthritis Safety Study (CLASS)^8^ was a large prospective randomised, double-blind trial with patients followed for up to 15 months evaluating GI outcomes using celecoxib and nonselective NSAIDs for the treatment of patients with arthritis. As haemoglobin values were collected sequentially over time in both trials, we identified an opportunity to evaluate changes in haemoglobin values in these two independent trials. We hypothesised that the pattern of haemoglobin decreases (presumably associated with chronic gross or occult blood loss) may be similar between the trials. Thus, in these two studies and in a non-aspirin using population, we evaluated and compared the incidence and time to event for a meaningful end point defined by a decrease in haemoglobin of ≥2 g/dL^18^ after 6 months. To ensure the focus of our results would better reflect clinical practice we also analysed patients in whom the ≥2 g/dL decrease in haemoglobin led to laboratory-defined anaemia (defined as a haemoglobin level ≤11.5 g/dL).

## Materials and methods

### Study designs

**Figure 1:**
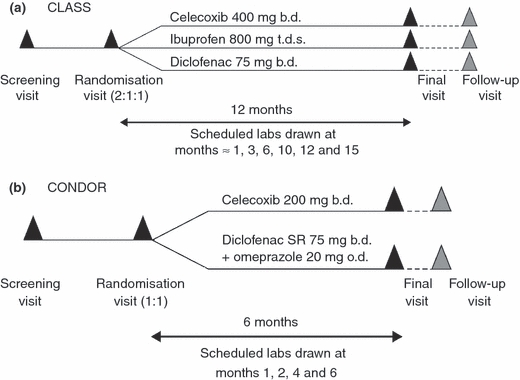
Study designs for the (a) CLASS and (b) CONDOR trials.

The CLASS trial was a double-blind, prospective, randomised clinical trial conducted in patients with OA and RA in North America. Patients were enrolled based on the judgement of the local principal investigator regardless of the patients’ baseline GI risk for an NSAID-associated complication. A total of 8059 patients were enrolled and 7968 randomised in a 2:1:1 ratio to receive oral celecoxib 400 mg b.d. (Pfizer Inc, New York, NY, USA), ibuprofen 800 mg t.d.s. (G.D. Searle & Co., Skokie, IL, USA), or diclofenac 75 mg b.d. (G.D. Searle & Co.) for up to 15 months (Figure 1a). Aspirin use for cardiovascular (CV) prophylaxis was allowed (≤325 mg/day) in this trial. The primary end point was the incidence of predefined adjudicated upper GI events as previously reported.^8^ In comparison, the CONDOR trial (NCT00141102) was a double-blind, triple-dummy, randomised, parallel-group, multicentre, international study comparing treatment with celecoxib (Pfizer Inc) alone versus diclofenac sustained release (SR) (Novartis Pharmaceuticals UK, Camberley, Surrey, UK) plus omeprazole (AstraZeneca LP, Wilmington, DE, USA) in patients with OA and/or RA at increased risk of GI adverse events. A total of 4484 patients were enrolled and randomised in a 1:1 ratio to receive either oral celecoxib 200 mg b.d. or diclofenac SR 75 mg b.d. plus omeprazole 20 mg o.d. for 6 months (Figure 1b). No aspirin use was allowed in this trial. The primary end point was the incidence of a predefined comprehensive composite primary end point of both upper and lower GI events, including clinically significant decreases in haemoglobin (≥2 g/dL) and/or haematocrit (≥10%), as previously reported.^16^, ^17^

### Study population

In the CLASS trial male or female patients aged ≥18 years were eligible for inclusion if they had been diagnosed with OA/RA for at least 3 months and were expected to require continuous treatment with an NSAID for the duration of the trial; patients who were *Helicobacter pylori* positive and/or using aspirin were not excluded from this trial. In the CONDOR trial, male or female patients with OA/RA who were expected to require daily prescription of anti-inflammatory analgesic therapy for the management of their arthritis symptoms and who had increased GI risk were eligible for inclusion. For the CONDOR trial, increased GI risk was defined as those patients aged ≥60 years or ≥18 years who had gastroduodenal (GD) ulceration 90 days or more prior to screening. In contrast to CLASS, patients who were *H. pylori* positive or using aspirin/other anti-platelet agents were excluded from the CONDOR trial. To make a parallel analysis in both trials, patients in the CLASS trial who were receiving aspirin (*n* = 882 and *n* = 445 in the celecoxib and diclofenac groups, respectively) were excluded from this analysis. Furthermore, since only diclofenac (SR) was used in CONDOR as the nonselective NSAID whereas diclofenac and ibuprofen were used in CLASS, we only evaluated the pattern of clinically significant decreases in haemoglobin ≥2 g/dL with diclofenac vs. celecoxib (Table 1). While we are comparing diclofenac arms in the two trials it should be noted that the formulations of diclofenac differed, as shown in Table 1. Furthermore, a substantial difference between these trials that may have affected this analysis was the required use of PPIs in the CONDOR trial which was not allowed in the CLASS trial. For this analysis we made the assumption that injury beyond the upper GI tract would not be influenced by the administration of a PPI.

**Table 1:** Comparison of the CLASS and CONDOR trials

CLASS	CONDOR
Similarities	
OA or RA	
Anticipated regular use of NSAID therapy for at least 6 months	
Use of NSAIDs, anti-ulcer drugs, sucralfate, misoprostol excluded	
Differences	
Up to 15 months duration	6 months duration
Age ≥18 years	Increased GI risk (age ≥60 years or ≥18 years with history of GD ulcer)
*H. pylori* negative or positive	*H. pylori* negative
Aspirin (≤325 mg) use allowed	No aspirin use
Celecoxib 400 mg b.d.	Celecoxib 200 mg b.d.
Diclofenac, ibuprofen; PPI use was not allowed	Diclofenac SR plus PPI (omeprazole)

OA, osteoarthritis; RA, rheumatoid arthritis; NSAID, nonsteroidal anti-inflammatory drug; PPI, proton pump inhibitor.

### End points

For both trials, a decrease in haemoglobin of ≥2 g/dL was used to define the clinically meaningful end point as previously described by us and others.^16^–^18^ In addition, a *post hoc* analysis was performed for patients who had a decrease in haemoglobin ≥2 g/dL that resulted in laboratory-defined anaemia (haemoglobin level ≤11.5 g/dL). While the CLASS trial followed patients for up to 15 months, for purposes of this analysis, we chose the 6-month time frame to allow us to make parallel comparisons between the two studies.

### Statistical analysis

The proportions of patients in the CLASS and CONDOR trials with a haemoglobin drop of ≥2 g/dL were estimated using the laboratory data collected at scheduled and unscheduled visits; odds ratio and confidence intervals (CI) have also been presented. The time to haemoglobin drop of ≥2 g/dL was analysed using a log-rank test and summarised using Kaplan–Meier curves; the proportion of patients with haemoglobin drops of ≥2 g/dL by day 180 (6 months) were also estimated from the Kaplan–Meier curve.

## Results

### Patient populations and demographics

**Figure 2:**
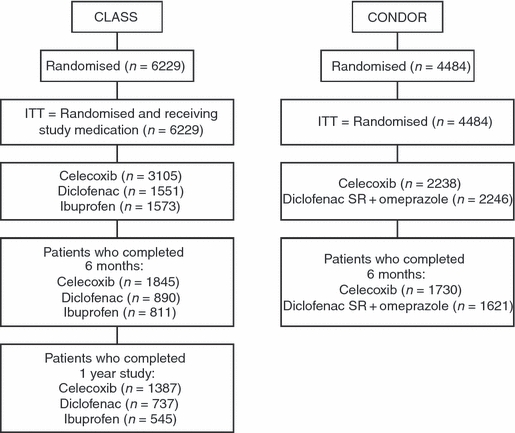
Study disposition (non-aspirin users in the CLASS and CONDOR trials).

Overall 1845 and 1730 non-aspirin users receiving celecoxib and 890 and 1621 non-aspirin users receiving diclofenac completed the CLASS and CONDOR trials, respectively (Figure 2). In CLASS, 41.3% of patients withdrew from the trial in the celecoxib and diclofenac arms by 6 months for reasons including treatment failure, protocol violation, noncompliance, laboratory abnormalities, adverse events and death (Table S1); in CONDOR, 25.3% withdrew for similar reasons (Table S2).^16^

For the purposes of this analysis, only the baseline demographics for those patients who were non-aspirin users in the CLASS trial are displayed (Table 2). The baseline demographics for the complete total population in CLASS have previously been reported by Silverstein *et al.*^8^

**Table 2:** Baseline demographics in non-aspirin users in the CLASS and CONDOR trials

	CLASS		CONDOR	
		
Characteristics	Celecoxib (*n* = 3105)	Diclofenac (*n*= 1551)	Celecoxib (*n* = 2238)	Diclofenac SR (*n* = 2246)
Women, %	71.2	70.0	82.6	81.1
Diagnosis, %				
OA	68.4	69.6	84.1	84.2
RA	26.4	25.3	15.9	15.8
OA & RA	5.2	5.1	–	–
Mean age, years (s.d.)	59.5 (11.7)	59.0 (12.0)	65.2 (7.8)	65.3 (7.6)
White, %	87.9	88.5	55.3	54.0
Previous history of GD ulcer, %	7.8	8.4	17.7	17.8
*H. pylori* infection*, %*	38.2	37.3	–	–
Haemoglobin (g/dL)	13.6 (1.3)	13.6 (1.3)	13.6 (1.1)	13.6 (1.1)

GD, gastroduodenal; OA, osteoarthritis; RA, rheumatoid arthritis.

There were more women and more patients with OA or a previous history of GD ulcers in the CONDOR trial than in CLASS, while more patients in the CLASS trial were white. Although patients with *H. pylori* infection at baseline were excluded from the CONDOR trial (patients had to test negative for *H. pylori* at the screening visit or to have confirmed eradication of the infection at a re-screening visit to be included), they were included in the CLASS trial; the patient population used in this secondary analysis included participants regardless of their *H. pylori* status. In the CLASS trial 38.2% and 37.3% of patients were *H. pylori* positive in the celecoxib and diclofenac treatment arms respectively (Table 2).

### CLASS and CONDOR: Patients meeting a ≥2 g/dL decrease in haemoglobin during 6 months

**Figure 3:**
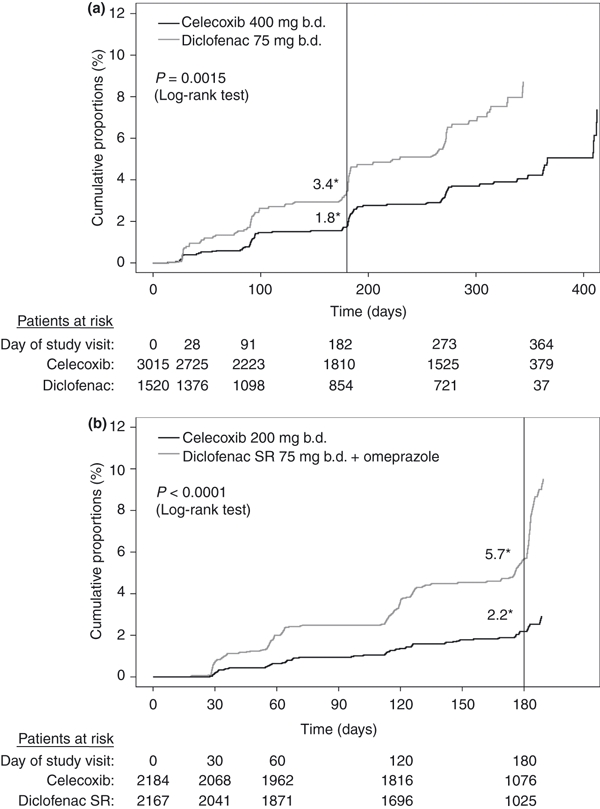
Kaplan–Meier summaries in non-aspirin patients with haemoglobin decreases (≥2 g/dL) in the (a) CLASS and (b) CONDOR trials. *Estimated proportion at month 6.

In the non-aspirin using population of the CLASS and CONDOR trials, 1.9% and 2.0% of patients treated with celecoxib developed a ≥2 g/dL decrease in haemoglobin during 6 months of treatment, respectively. In contrast, a greater percentage of patients receiving diclofenac developed a ≥2 g/dL decrease in haemoglobin by 6 months; 3.3% in CLASS and 5.7% in CONDOR, respectively. The corresponding odds ratio for CLASS and CONDOR were both significant at 6 months [CLASS: 1.80 (95% CI, 1.22–2.65) and CONDOR: 2.93 (95% CI, 2.06–4.15), respectively]. As seen in Figure 3a for CLASS and 3b for CONDOR, the time to a decrease in haemoglobin ≥2 g/dL occurs at a slower rate in the celecoxib arm than in the diclofenac arm (log-rank test *P* < 0.01). Furthermore, based on these Kaplan–Meier curves (Figure 3a,b), the estimates of the proportion of patients with a decrease in haemoglobin ≥2 g/dL were 1.8% and 3.4% in CLASS and 2.2% and 5.7% in CONDOR for the celecoxib and diclofenac arms, respectively. Despite differences in the methodology for calculating these rates (incidence vs. Kaplan–Meier analysis), the rates reported above are consistent with the Kaplan–Meier curves in Figure 3a,b.

### Aspirin users crude rate

In the CLASS trial, a sub-population of aspirin users (≤325 mg per day of aspirin) was identified.^8^ As seen in Table 3, at 6 months there was a greater percentage of subjects with a decrease in haemoglobin ≥2 g/dL in those using aspirin plus diclofenac as compared to those receiving aspirin plus celecoxib with a corresponding odds ratio of 1.71 (95% CI, 1.00–2.92).

**Table 3:** Aspirin users in CLASS (crude rates over 6 months)

	No decrease in haemoglobin, *n* (%)	With a decrease in haemoglobin, *n* (%)
Celecoxib *N* = 872	841 (96.44)	31 (3.56)
Diclofenac *N* = 438	412 (94.06)	26 (5.94)
	OR = 1.71 (1.00–2.92)	

### CLASS and CONDOR: Patients meeting a ≥2 g/dL decrease in haemoglobin and meeting laboratory-defined anaemia criteria during 6 months

We have further evaluated the decrease in haemoglobin in both trials using a singular absolute cut-off value at 6 months of ≤11.5 g/dL, the laboratory-defined lower limit of normal (LLN) for haemoglobin values in the CONDOR trial (but not the CLASS trial – please refer to text below). Among the celecoxib patients who had a decrease in haemoglobin ≥2 g/dL, 21.4% (12/56) of patients in CLASS and 18.2% (8/44) of patients in CONDOR had a final haemoglobin value ≤11.5 g/dL. In contrast, in the diclofenac arms 16.0% (8/50) of patients in CLASS and 51.2% (63/123) of patients in CONDOR who had a decrease in haemoglobin ≥2 g/dL had a final haemoglobin value ≤11.5 g/dL.

As the LLN for haemoglobin values were different for men (<12.7 g/dL for men aged 12–59 years and <12.5 g/dL for men aged 60–150 years) and women (<11.6 g/dL for women aged 12–59 years and <11.5 g/dL for women aged 60–150 years) in the CLASS trial, we further calculated decreases in haemoglobin ≥2 g/dL in men and women separately. As such, in the CLASS trial the odds ratio for patients having decreases in haemoglobin ≥2 g/dL was 1.63 for women (95% CI, 0.98–2.72) and 2.06 men (95% CI, 1.14–3.74) (Table S3).

## Discussion

Our study demonstrates that continuous 6-month exposure to NSAIDs is associated with a clinically meaningful decrease in haemoglobin; a meaningful decline in haemoglobin was observed in all of the treatment arms in both trials in the absence of aspirin use. However, despite differences in the study design and population of the two trials, these results were reproducible for both. Furthermore, the studies demonstrated that patients treated with diclofenac were two- to threefold more likely to reach the meaningful end point than patients treated with the COX-2 selective NSAID celecoxib. We believe that this information focused on the development of progressive haemoglobin decreases with the chronic use of NSAIDs, independent of acute bleeding events, and highlights the relatively understudied clinical issue of long-term chronic occult blood loss.

A decrease in haemoglobin of ≥2 g/dL has previously been considered as a clinically meaningful end point^18^ in the evaluation of GI toxicity in NSAID users. Presumably, this assumes a decrease in haemoglobin of ≥2 g/dL is reflective of overt or occult blood loss and represents a level of decline that is clinically meaningful and could prompt physicians to intervene with new clinical decisions and/or interventions. While we believe that such decreases in haemoglobin can impact clinical care, adverse outcomes potentially attributed to declines in haemoglobin are not captured in either study.

Based on these considerations, this practical end point has previously been utilised as an important outcome in a number of large GI outcome trials evaluating NSAID-associated toxicity such as VIGOR (Vioxx Gastrointestinal Outcomes Research),^4^ CLASS,^8^ TARGET (Therapeutic Arthritis Research and Gastrointestinal Event Trial)^6^ and MEDAL (Multinational Etoricoxib and Diclofenac Arthritis Long-term).^5^ Moreover, the direct clinical relevance of this end point and, notably the development of anaemia, are further supported in the literature as being associated with a significant increased risk of recurrent falls, hospitalisations and mortality, particularly in older adults.^19^–^22^ In addition, anaemia or occult blood loss may result in diminished physical performance and a lesser quality of life.^23^–^25^ Low haemoglobin levels are now recognised as an important issue that affects a number of clinical patient outcomes – in particular, the clinical impact of anaemia in the elderly population has been clearly documented.^19^–^22^

Both the CLASS (conducted from 1998–2000) and CONDOR (conducted from 2005–2009) trials were designed to assess NSAID-related damage in the GI tract in patients with undefined or increased GI risk, respectively. However, there were a number of differences between the two trials that potentially affected the findings (Table 1). One of the key differences was that in CLASS patients were randomised to diclofenac alone whereas, in CONDOR patients in the comparator group were randomised to diclofenac SR plus a PPI (omeprazole 20 mg o.d.). While the addition of a PPI in the CONDOR trial would be expected to reduce the impact of diclofenac on the upper GI tract with little or no effect in the lower GI tract, the rate of defined upper GI events was still higher in the diclofenac plus PPI arm than in the celecoxib arm.^16^ However, overall the rate of upper GI events was relatively small and thus, we believe the majority of the meaningful blood loss end points were not driven by any small differences in upper GI outcomes but instead are likely to reflect small bowel occult blood loss over time with continuous NSAID exposure. Presumably this differential rate might be reflected by the differences in injury patterns in the small bowel following use of nonselective and COX-2 selective NSAIDs.^12^, ^13^, ^26^

In the development of this *post hoc* analysis, differences in the design of the two clinical trials excluded the possibility for a pooled analysis. Patients in the CLASS trial were on average younger than those in the CONDOR trial [mean age 59.1 years (±s.d. 6.9) vs. 66.4 years (±s.d. 6.9), respectively], reflecting an increased level of GI risk for patients in the CONDOR trial. There were also differences in the dosing of medications between the two trials; in the CLASS trial a higher dose of celecoxib 400 mg b.d. was used compared with celecoxib 200 mg b.d. in the CONDOR trial. In addition, as this was a non-US trial and diclofenac SR is one of the most popular NSAIDs used outside the United States, this formulation was used in the CONDOR trial. However, it is theoretically possible that the diclofenac SR formulation used in the CONDOR trial may have been associated with a differing degree or location of intestinal injury as compared to the diclofenac formulation in the CLASS trial, although there is a lack of any head-to-head comparisons between the two formulations in the literature regarding small bowel injury.

Furthermore, in the CLASS trial both *H. pylori* positive and negative patients were included in the analysis, whereas in CONDOR patients who tested positive for *H. pylori* were excluded from the study (unless they were specifically treated for their infection and subsequently tested negative). As seen in Figure 1, over the first 6 months of the CLASS and CONDOR trials the frequency of scheduled laboratory evaluations differed. While CLASS subjects had three evaluations after baseline, in the CONDOR trial there were four post-baseline evaluations scheduled. As such the frequency of laboratory evaluations might have influenced the rate of events observed in the CONDOR trial as compared with CLASS (Figure 3a,b).

We made several speculations in the analysis of these data. While we believe the reported haemoglobin changes are due to GI blood loss, we have no data regarding occult blood loss to prove this is true. Moreover, there were also patients embedded in the population who had other reasons for the decrease in haemoglobin seen over time. Of those cases reaching the predefined ≥2 g/dL decrease in haemoglobin component of the primary end point in the CONDOR population, 32% of patients were adjudicated as having a non-GI aetiology due to other causes (e.g. flare of their underlying rheumatological disorder).

Although we have used this objective end point to evaluate changes in haemoglobin, as suggested by the US Food and Drug Administration (FDA) and used by others, we did not directly evaluate medical outcomes associated with the development of this meaningful drop in haemoglobin. These end points, such as recurrent falls, hospitalisations and mortality, would not be expected to occur to a significant degree over the limited time frame of this study and given the limited number of patients.

Based on our 6-month data as shown above, and in Figure 3a,b, there is a discrepancy between the trials in the relative number of patients achieving the laboratory-defined (≤11.5 g/dL) anaemia end point. In CONDOR a significantly greater number of patients in the diclofenac group had a ≥2 g/dL decrease in haemoglobin and a haemoglobin value ≤11.5 g/dL during 6 months. However, in CLASS this difference was not apparent. Interestingly the difference between the two trials is not readily explained by a difference in the mean haemoglobin values for the two populations at baseline. In the CLASS trial, the mean haemoglobin values at the onset of the study were 13.6 (s.d. 1.3) in the celecoxib and diclofenac treatment arms, respectively. In the CONDOR trial the mean haemoglobin values at baseline were 13.6 (s.d. 1.1) for both treatment arms (Table 2). Given the small number of patients achieving haemoglobin values ≤11.5 g/dL in the CLASS trial it would be imprudent to draw any immediate conclusions regarding the development of anaemia in this trial.

Despite its limitations we believe this study provides important clinical findings. Decreases in haemoglobin are clinically meaningful in that they drive medical evaluations and increased cost of care – the use of the absolute cut-off of ≥2 g/dL has been advocated by the FDA^18^ and supported by the European Medicines Agency (EMA), among other researchers.

In these two large-scale, randomised and independent outcome trials clinically meaningful decreases in haemoglobin ≥2 g/dL occurred in a similar fashion over time despite differences in trial design. Among non-aspirin using populations, a significantly greater number of patients treated with diclofenac had reductions in haemoglobin as compared with patients treated with celecoxib over 6 months. Interestingly, the difference between these two arms in CLASS, in both the aspirin and non-aspirin using population, is consistent with numerically similar odds ratios.

We believe that the reproducibility of these findings over 6 months confirms their clinical importance in this age-relevant population with arthritis and that these data will provide the foundation for future research regarding the effects of anti-inflammatory agents throughout the entire GI tract.

## SUPPORTING INFORMATION

**Table S1.** Reasons for withdrawing from the CLASS trial in non-aspirin users.

**Table S2.** Reasons for withdrawing from the CONDOR trial in non-aspirin users.

**Table S3.** Incidence of clinically significant haemoglobin decreases (≥2 g/dL) up to month 6 in non-aspirin users in the CLASS trial by sex.
