# Supplementary material for: Haemoglobin decreases in NSAID users over time: an analysis of two large outcome trials
Source: Aliment Pharmacol Ther. 2011 Aug 2;34(7):808–16. doi: 10.1111/j.1365-2036.2011.04790.x (PMC3201839; doi:10.1111/j.1365-2036.2011.04790.x)
Supplement: Supplementary file 1 [file apt0034-0808-SD1.doc]

**Table S1. Reasons for withdrawing from the CLASS trial in non-aspirin users**

|  | **CLASS** | | |
| --- | --- | --- | --- |
|  | **Celecoxib**  **(*n* = 3105)** | **Diclofenac**  **(*n* = 1551)** | **Ibuprofen**  **(*n* =1573)** |
| **Withdrawal (all causes), *n* (%)** | 1718 (55.3) | 814 (52.5) | 1028 (65.4) |
| Treatment failure | 555 (17.9) | 243 (15.7) | 361 (22.9) |
| Pre-existing violation | 19 (0.6) | 7 (0.5) | 11 (0.7) |
| Non compliance | 477 (15.4) | 162 (10.4) | 298 (18.9) |
| Adverse events | 667 (21.5) | 402 (25.9) | 358 (22.8) |

**Table S2. Reasons for withdrawing from the CONDOR trial in non-aspirin users**

|  | **CONDOR** | |
| --- | --- | --- |
|  | **Celecoxib**  **(*n* = 2238)** | **Diclofenac SR**  **(*n* = 2246)** |
| **Withdrawal (all causes), *n* (%)** | 508 (22.7) | 625 (27.8) |
| Did not receive treatment | 15 (0.7) | 9 (0.4) |
| Discontinued treatment | 493 (22.0) | 616 (27.4) |
| Adverse events | 233 (10.4) | 305 (13.6) |
| Laboratory abnormalities | 9 (0.4) | 33 (1.5) |
| Death | 2 (0.1) | 2 (0.1) |
| Other | 249 (11.1) | 276 (12.3) |

**Table S3. Incidence of clinically significant haemoglobin decreases (≥2 g/dL) up to month 6 in non-aspirin users in the CLASS trial by sex**

|  | **CLASS (FEMALE)** | | **CLASS (MALE)** | |
| --- | --- | --- | --- | --- |
|  | **Number of patients** | **Patients with a haemoglobin decrease  (≥2 g/dL)** | **Number of patients** | **Patients with a haemoglobin decrease  (≥2 g/dL)** |
| **Celecoxib, n (%)** | 2210 | 34 (1.54) | 895 | 22 (2.46) |
| **Diclofenac, n (%)** | 1085 | 27 (2.49) | 466 | 23 (4.94) |
| **Odds ratio (95% CI)** | 1.63  (0.98–2.72) | | 2.06  (1.14–3.74) | |
